# Transcriptional response of *Bacillus megaterium* FDU301 to PEG200-mediated arid stress

**DOI:** 10.1186/s12866-020-02039-4

**Published:** 2020-11-16

**Authors:** Lei Zhao, Yanjun Zhou, Jianbei Li, Yucheng Xia, Weiyun Wang, Xiuqi Luo, Juan Yin, Jiang Zhong

**Affiliations:** 1 Department of Microbiology and Microbial Engineering and State Key Laboratory of Genetic Engineering, School of Life Sciences, Fudan University, Shanghai, 200438 China; 2 Institute for Preservation and Conservation of Chinese Ancient Books, Fudan University, Shanghai, 200433 China

**Keywords:** Arid stress, *Bacillus megaterium*, Polyethylene glycol 200, Transcriptome, RT-qPCR

## Abstract

**Background:**

For microorganisms on a paper surface, the lack of water is one of the most important stress factors. A strain of *Bacillus megaterium* FDU301 was isolated from plaques on a paper surface using culture medium with polyethylene glycol 200 (PEG200) to simulate an arid condition. Global transcriptomic analysis of *B. megaterium* FDU301 grown under normal and simulated arid conditions was performed via RNA-seq technology to identify genes involved in arid stress adaptation.

**Results:**

The transcriptome of *B. megaterium* FDU301 grown in LB medium under arid (15% PEG200 (w/w)) and normal conditions were compared. A total of 2941 genes were differentially expressed, including 1422 genes upregulated and 1519 genes downregulated under arid conditions. Oxidative stress-responsive regulatory genes *perR*, *fur*, and *tipA* were significantly upregulated, along with DNA protecting protein (*dps*), and catalase (*katE*). Genes related to Fe^2+^ uptake (*feoB*), sporulation stage II (*spoIIB*, *spoIIE*, *spoIIGA*), small acid-soluble spore protein (*sspD*), and biosynthesis of compatible solute ectoine (*ectB*, *ectA*) were also highly expressed to various degrees. Oxidative phosphorylation-related genes (*atpB, atpE, atpF, atpH, atpA, atpG*, *atpD*, *atpC*) and glycolysis-related genes (*pgk*, *tpiA*, *frmA*) were significantly downregulated.

**Conclusion:**

This is the first report about transcriptomic analysis of a *B. megaterium* to explore the mechanism of arid resistance. Major changes in transcription were seen in the arid condition simulated by PEG200 (15%), with the most important one being genes related to oxidative stress. The results showed a complex mechanism for the bacteria to adapt to arid stress.

**Supplementary Information:**

The online version contains supplementary material available at 10.1186/s12866-020-02039-4.

## Background

Microorganisms are affected by various environmental factors, and successful adaptation to these factors is key for microbial colonization. Arid stress is caused by a lack of water or by high concentration of salts in the environment. However, microbes living in arid deserts, drying foods and plant rhizosphere have developed complicated strategies to survive under arid conditions [1–3].

The water available to living organisms in a sample can be represented by water activity (aw), which is the ratio between the vapor pressure of the sample, and that of distilled water, under the same conditions [4]. Microorganisms require a certain minimum level of water activity to grow normally [5].

Suitable water activity is important for fungal cell morphology and biochemical responses [5, 6]. Under arid stress, the differentiation and division of fungal cells slow down and the metabolism is significantly suppressed [7, 8]. *Aspergillus* spp. and *Saccharomyces cerevisiae* synthesize and accumulate compatible solutes, such as glycerol, to protect proteins and nucleic acids [9–11]. In response to the lack of water, *Xeromyces bisporus* and some other food-borne molds have increased amount of saturated fatty acid in their membrane, and form extracellular polymers [12].

Compared to fungi, most bacteria are less adaptable to arid stress. Studies on the adaptation of bacterial to arid condition have mostly been on food-borne pathogens [13, 14] and soil bacteria [15, 16]. *Pseudomonas* spp. respond to arid stress by secreting polysaccharides and changing the composition of fatty acid to maintain membrane fluidity [15]. Bacteria also synthesize or uptake compatible solutes, such as glycine-betaine, proline, trehalose and ectoine to resist arid stress [17, 18]. For example, the upregulation of osmoprotectant transporters (*proU*, *osmU*, *proP*) and trehalose biosynthetic genes were found during the *Salmonella* arid tolerance [19, 20]. Alternative sigma factors (for example, *rpoS*) are essential for coordinating *S. enterica* adaptation to arid stress [21]. In addition, iron-sulfur (Fe-S) cluster synthesis-related genes (such as *nifU*, *nifS*, *iscA*, *sufD*), and virulence factors (*sopD* and *sseD*) are essential for the survival of *Salmonella* in arid environments [13, 22–25].

Arid stress is known to increase the formation of reactive oxygen species (ROS) in bacteria, leading to lipid peroxidation, protein denaturation and nucleic acid damage [26]. It has been shown that bacteria adapt to the oxidative stress caused by arid environments by accumulating intracellular Mn^2+^, which is involved in protein protection [27]. SigB activity plays an important role in *Staphylococcus aureus* adaptation to oxidative stress caused by arid environments [28].

There are relatively few studies on the arid tolerance of *Bacillus*. In general, *Bacillus* tolerate arid and osmotic stress by rapidly accumulating compatible solutes or opening channels for ions such as Na^+^ and K^+^ [29]. Forming spore is also a common strategy of *Bacillus* to survive adverse environments. The transcriptomic response to arid conditions has not been well studied in *Bacillus*.

In this study, we used polyethylene glycol 200 (PEG200) to simulate arid stress in the medium [30], since PEG200 had no direct role in bacterial physiology and metabolism. A strain of *B. megaterium* FDU301 adaptable to arid conditions was isolated from a paper surface. Using transcriptome technology (RNA-seq), the gene expressions of *B. megaterium* FDU301 under simulated arid and normal conditions were compared. This work is aimed at offering a new perspective to understand the adaptation of bacteria on paper surface, and to control bacteria-related deterioration of paper documents in the future.

## Results

### Characterization of *B. megaterium* FDU301

A strain of *B. megaterium* tolerant to arid condition (15% PEG200 (w/w), aw 0.985) was isolated from plaque areas on the surface of a leaflet in an old book, and was named as FDU301. The sequence of its 16S rDNA gene was identical to that of *B. megaterium* NBRC15308 and *B. megaterium* QMB1551 (data not shown). The whole genome of *B. megaterium* FDU301 was sequenced on a combination of Illumina HiSeq and PacBio RSII platforms [31]. The assembled genome was 6,872,701 bp in length, comprising one chromosome and nine plasmids.

Comparing with *B. megaterium* NBRC15308, FDU301 had a larger genome, and more predicted genes. Under the conditions of amino acid sequence identity being greater than 40% over at least 80% of the full sequence length [32], FDU301 and NBRC15308 had 5335 homologous genes. Among 1561 genes unique to FDU301, 305 genes were annotated with KEGG database to be related to signaling and cellular processes, environmental information processing, genetic information processing, carbohydrate metabolism, etc. The full genome data of *B. megaterium* FDU301 can be found in NCBI GenBank (accession numbers CP045267-CP045276).

**Fig. 1:**
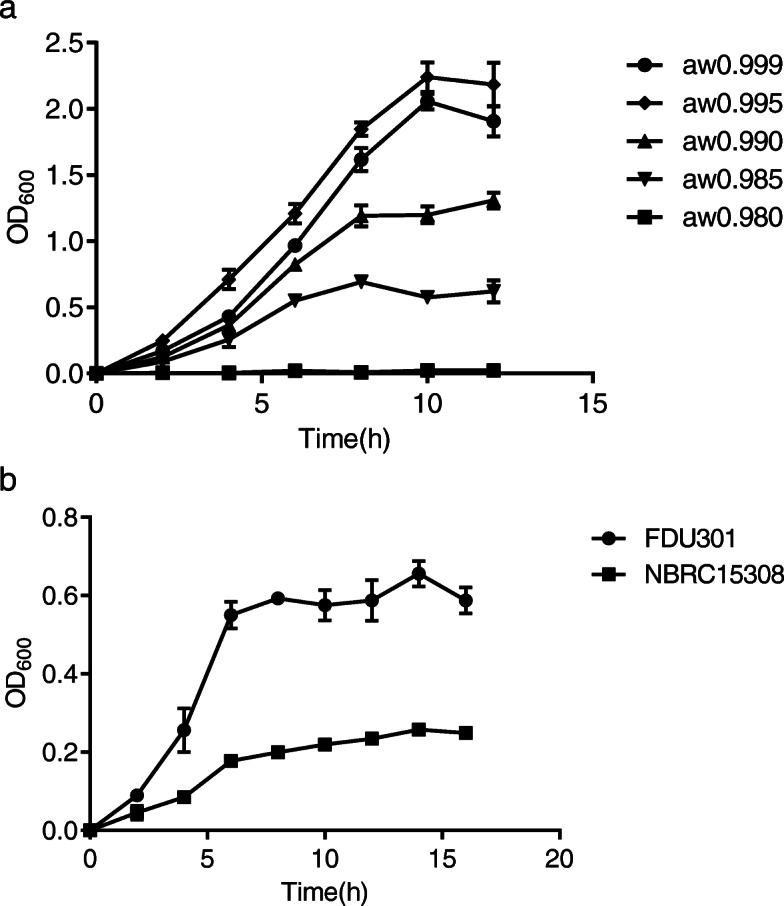
Growth curves of *B. megaterium* FDU301 and *B. megaterium* NBRC15308. **a**: Grwoth of *B. megaterium* FDU301 in LB medium with different concentration of PEG200. **b**: Grwoth of *B. megaterium* FDU301 and *B. megaterium* NBRC15308 in LB medium with 15% PEG200

As shown in Fig. 1a, FDU301 showed a typical “S” type growth curve in normal LB medium, with an incubation period of 2 h, and reached a plateau around 10 h. In the presence of 5% PEG200, the FDU301 grew faster and reached higher cell density than that in normal LB medium. As the concentration of PEG200 increased, the growth of bacteria slowed down and reached much lower cell density than that in normal LB medium. The bacteria hardly grew in the medium with 20% PEG200, indicating the limit of the strain to tolerate. Compared to *B. megaterium* NBRC15308, *B. megaterium* FDU301 grew much better in the arid medium (15% PEG200 (w/w)) (Fig. 1b).

### Global overview of the RNA-Seq data

The transcriptome of *B. megaterium* FDU301 in the growth phase (4 h) under control (LB medium, L) and the simulated arid condition (LB medium with 15% PEG200, P) was analyzed with RNA-seq. The RNA-seq data have been submitted to NCBI SRA (accession numbers PRJNA649685). After filtration, a total of 51,893,124 and 61,892,804 reads were obtained from L and P samples, respectively. For both samples, more than 95% of the reads were mapped to the *B. megaterium* FDU301 reference genome (Table 1). In order to verify the transcriptomic results, ten differentially expressed genes (DEGs) were randomly selected and their transcriptional level were determined with quantitative reverse transcription PCR (RT-qPCR). The results of RNA-seq and RT-qPCR were generally consistent with each other, indicating that the transcriptomic results reflected the differences in gene expression under the arid and normal conditions (Additional file 1: Figure S1).

**Table 1:** Quality control statistics of transcriptomic data and genome mapping

Sample name	Clean reads (bp)	Clean bases (bp)	Q30 (%)	Genome mapped reads (bp)	Genome mapped ratio (%)
L_1	18,048,182	2,440,791,296	95.54	17,618,940	97.62
L_2	17,345,958	2,344,077,298	95.46	16,977,526	97.88
L_3	16,498,984	2,197,149,343	95.30	16,090,245	97.52
P_1	21,088,294	2,798,604,312	95.48	20,290,912	96.22
P_2	18,406,784	2,415,364,313	95.71	17,735,307	96.35
P_3	22,397,726	2,886,319,033	95.76	21,341,293	95.28

**Fig. 2:**
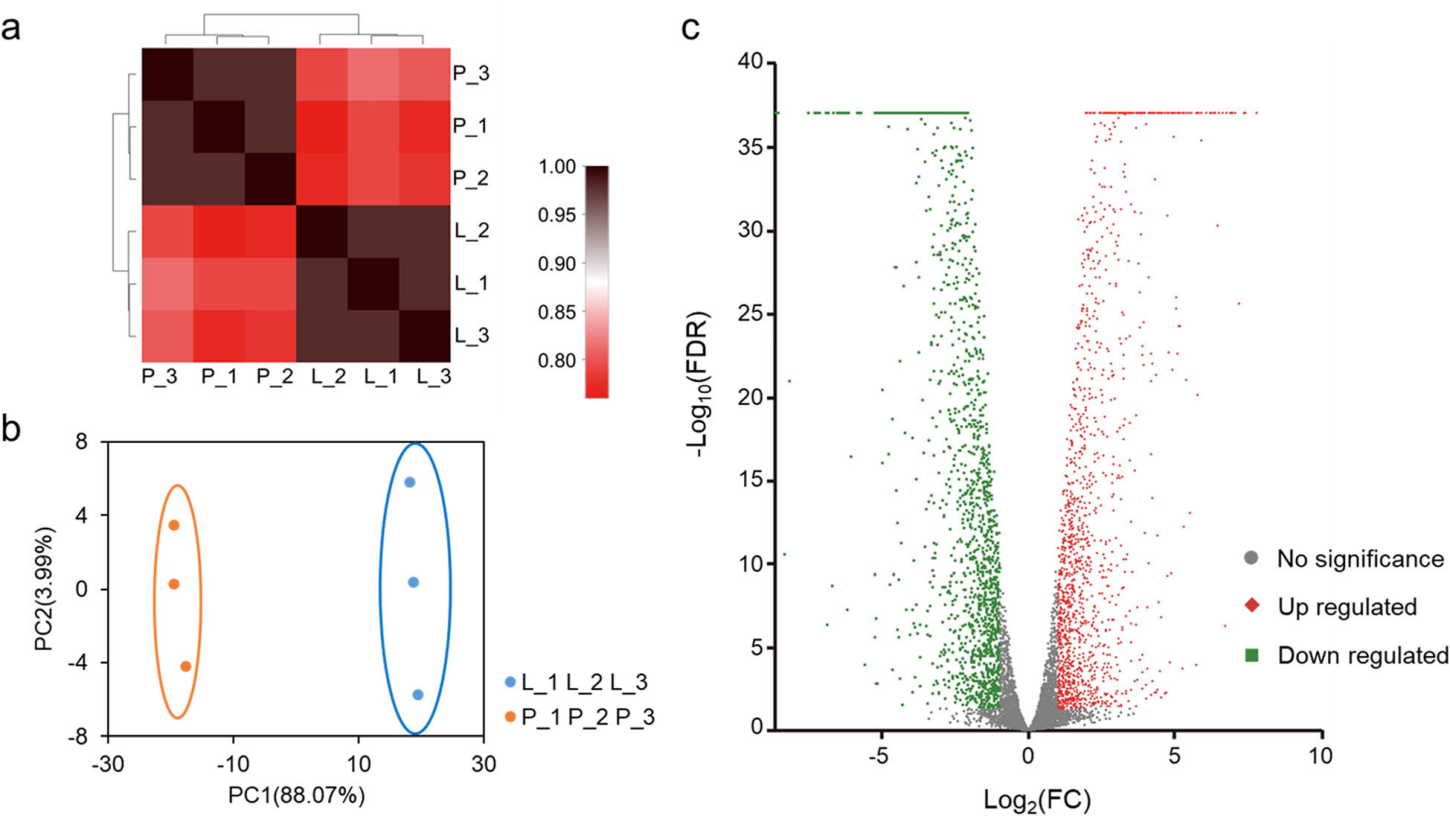
Transcriptomic analysis of *B. megaterium* FDU301 cultured for 4 h. **a**: Heatmap of the gene expression correlation, **b**: The results of PCA analysis, **c**: Volcano map of differentially expressed genes. L_1, L_2, L_3 and P_1, P_2, P_3 were replicas of group L (LB medium) and P (LB medium with 15% PEG), respectively. Gray circles, red diamonds, and green represented genes with no significant changes, upregulated genes, and downregulated genes, respectively. FC: fold of change in the transcriptional level in the simulated arid condition (LB medium with 15% PEG200, w/w) comparing to normal LB medium

As shown in Fig. 2a, the correlation between the three biological replicates of each sample (L and P) was high, indicating that the sequencing data was highly reproducible. Meanwhile the difference between treatment groups was obvious. Two groups were also well separated from the other in the principal component analysis (Fig. 2b). These showed that arid stress had a significant effect on the gene expression of FDU301.

The volcano map of DEGs is shown in Fig. 2c. Compared with the control group, the expression levels of 2941 genes were significantly different under the simulated arid conditions (FDR < 0.05 & |log_2_FC| ≥ 1), of which 1422 genes were upregulated and 1519 genes were downregulated (Additional file 2: Table S1).

### Annotation analysis of DEGs

**Fig. 3:**
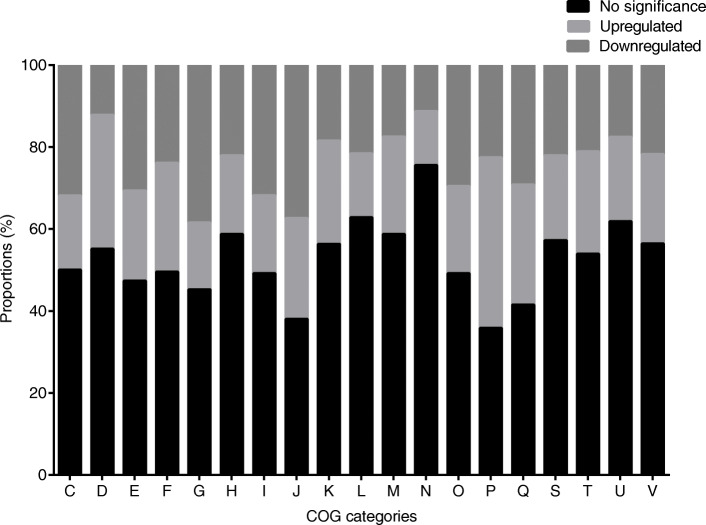
COG analysis of DEGs of *B. megaterium* FDU301 under normal and arid conditions. Proportions of all genes annotated with COG database that were upregulated (light grey), downregulated (dark grey), and not-significantly-changed (black) under the simulated arid condition (LB medium with 15% PEG200) comparing to normal condition (LB medium) were shown. C: energy production and conversion, D: cell cycle control, cell division, chromosome partitioning, E: amino acid transport and metabolism, F: nucleotide transport and metabolism, G: carbohydrate transport and metabolism, H: coenzyme transport and metabolism, I: lipid transport and metabolism, J: translation, ribosomal structure and biogenesis, K: transcription, L: replication, recombination and repair, M: cell wall/membrane/envelope biogenesis, N: cell motility, O: posttranslational modification, protein turnover, chaperones, P: inorganic ion transport and metabolism, Q: secondary metabolites biosynthesis, transport and catabolism, S: function unknown, T: signal transduction mechanisms, U: intracellular trafficking, secretion, and vesicular transport, V: defense mechanisms

The 2941 DEGs (FDR < 0.05 & |log_2_FC| ≥ 1) were annotated with COG and KEGG databases. According to COG annotation, DEGs were seen in most of the COG categories, which meant that the response of FDU301 to the arid stress was a complicated process (Additional file 3: Table S2). As shown in Fig. 3, the category with the highest proportion of upregulated genes was inorganic ion transport and metabolism (P, 41.56%). In terms of downregulated genes, categories with more than 30% genes downregulated included carbohydrate transport and metabolism (G); translation, ribosomal structure and biogenesis (J); energy production and conversion (C); lipid transport and metabolism (I); and amino acid transport and metabolism (E). These results suggested FDU301 had general suppressions in metabolism and protein production, and enhancement in the transport for inorganic ion, such as Fe, Zn, Ni, in face of the arid stress.

KEGG annotation showed that the arid stress induced a significant upregulation in the transcription of ABC transporters-associated genes (Additional file 4: Table S3), and a significant downregulation in that of oxidative phosphorylation and glycolysis-related genes (Additional file 5: Table S4).

### Major changes in gene expression under arid stress

Selected genes with significant changes in transcription under the simulated arid (15% PEG200 (w/w)) and normal conditions were further analyzed with RT-qPCR.

#### Oxidative stress-responsive genes

**Fig. 4:**
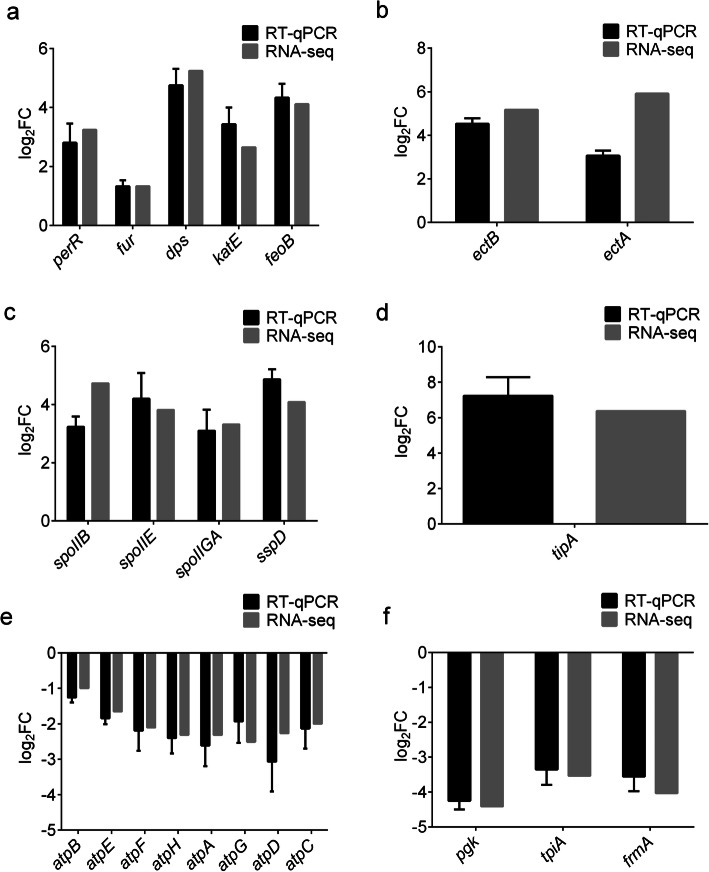
Changes in the expression of genes of *B. megaterium* FDU301 under normal and arid conditions. The transcription levels of selected genes were determined by transcriptomic analysis and RT-qPCR. **a**: Oxidative stress-responsive genes and Fe^2+^ transportation-related genes, **b**: Ectoine biosynthesis-related genes, **c**: Sporulation-related genes, **d**: *TipA*, **e**: Oxidative phosphorylation-related genes, **f**: Glycolysis-related genes. FC: fold of change in the transcriptional level in the simulated arid condition (LB medium with 15% PEG200, w/w) comparing to normal LB medium

PerR is a key regulatory protein for oxidative stress response in *Bacillus* spp. [33]. As shown in Fig. 4a, *perR* was upregulated under arid condition. Several genes known to be regulated by *perR* were also upregulated significantly, including *fur*, *dps*, and *katE* (Fig. 4a). *Fur* encodes a major suppressor for the expression of many ferrous uptake operons, whereas *dps* and *katE* are related to avoiding DNA damage and removing ROS, respectively. These results were consistent with the level ROS in FDU301 cells grown in the medium with different concentration of PEG200 (Additional file 6: Figure S2), suggesting that oxidative stress was one of the main challenges for the bacteria in the simulated arid condition.

#### Fe^2+^ transportation genes

Under oxidative stress, Fe^2+^ will react with H_2_O_2_ through fenton reaction to form hydroperoxide, which is highly active in destroying DNA. Upregulations of many Fe^2+^ transportation-related genes were seen in *B. megaterium* FDU301 under arid condition. In fact, Fur is a suppressor for Fe^2+^ uptake, and Dps functions by binding Fe^2+^ and reduces the level of free Fe^2+^ in the cell. Meanwhile, the Fe^2+^ uptake gene *feoB* [34], was also found to be greatly upregulated in arid condition (Fig. 4a), suggesting a delicate balance of Fe^2+^.

#### Ectoine biosynthesis genes

The *ectB* and *ectA* were significantly upregulated by about 23.10-fold and 8.40-fold, respectively in arid condition (Fig. 4b). The two genes are involved in the biosynthesis of compatible solute ectoine [35]. Meanwhile, genes related to the transportation and biosynthesis of another commonly used compatible solute, glycine-betaine, were not significantly changed, or even downregulated (Table 2, Additional file 2: Table S1).

**Table 2:** Changes of expression of *proVWX* operons under normal and arid conditions (RNA-seq)

Gene ID	Gene name	Description	Log_**2**_FC	FDR
FDZ14_RS20255	*proX*	glycine/betaine ABC transporter	−0.76	0.18
FDZ14_RS20265	*proV*	glycine/betaine ABC transporter ATP-binding protein	0.70	0.05
FDZ14_RS20260	*proW*	glycine/betaine ABC transporter	0.99	0.08
FDZ14_RS23515	*proX*	glycine/betaine ABC transporter	−0.92	0.00
FDZ14_RS23520	*proV*	glycine/betaine ABC transporter ATP-binding protein	0.42	0.07
FDZ14_RS23525	*proW*	glycine/betaine ABC transporter	0.74	0.00

#### Sporulation genes

Under the simulated arid condition, genes related to sporulation stage II (*spoIIB*, *spoIIE*, *spoIIGA*) were upregulated by about 8.57 to 18.38-fold. *SspD*, the gene encoding small acid-soluble spore proteins (SASP), which is a major protective component of *Bacillus* spores, were also highly expressed (Fig. 4c).

#### TipA gene

*TipA* encodes a transcriptional regulator activated by cyclic thiopeptide antibiotics, such as thiostrepton, and promothiocin in *Streptomyces* [36]. Under the simulated arid condition, *tipA* was one of the most dramatically upregulated genes, in terms of the fold of change in the transcriptional level (Fig. 4d). *TipA* had not been noticed in previous studies on bacterial arid tolerance.

#### Respiratory and glycolysis genes

As shown in Fig. 4e, under the simulated arid condition, genes related to oxidative phosphorylation (*atpB, atpE, atpF, atpH, atpA, atpG*, *atpD*, *atpC*) were downregulated to various degrees. Meanwhile, some glycolysis-related genes (*pgk, tpiA, frmA*) were also downregulated (Fig. 4f). This might reflect the slow growing status of bacteria in the simulated arid condition.

### Changes in gene expression under 5% PEG200

**Fig. 5:**
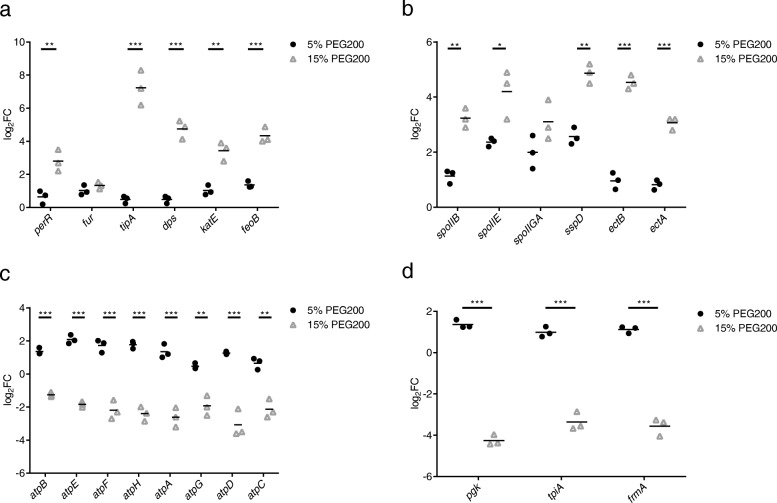
Changes in the expression of genes of *B. megaterium* FDU301 under different concentration of PEG200. The transcription levels of selected genes were determined by RT-qPCR. **a**: Oxidative stress-responsive genes, *tipA* and Fe^2+^ transportation-related genes, **b**: Genes related to ectoine biosynthesis and sporulation, **c**: Oxidative phosphorylation-related genes, **d**: Glycolysis-related genes. FC: fold of change in the transcriptional level in LB medium with 5% or 15% PEG200 comparing to normal LB medium. *: *p* < 0.05, **: *p* < 0.01, ***: *p* < 0.001

As shown in Fig. 1a, FDU301 grew slightly faster in medium with 5% PEG200 than in LB medium without PEG200. We compared the expression of selected genes with 0, 5, and 15% PEG200. Although genes upregulated in 15% PEG200 were also upregulated in 5% PEG200, the levels of upregulation for most of them were significantly lower than that in 15% PEG200 (Fig. 5a and b). In contrast, genes downregulated in 15% PEG200, including those in oxidative phosphorylation (*atpB*, *atpE*, *atpF*, *atpH*, *atpA*, *atpG*, *atpD*, *atpC*) and glycolysis (*pgk*, *tpiA*, *frmA*) pathways, were instead slightly upregulated (Fig. 5c and d). It seemed that under 5% PEG200, the bacteria sensed the change and increased their metabolisms to be prepared for worsen environments.

## Discussion

Most studies on the microorganisms of paper surfaces have focused on fungi [37, 38], which is generally more resistant to water deficiency and more harmful to paper than bacteria. However, the role of bacteria and their mechanisms to adapt to the environment also need to be explored, since recent high-throughput sequencing studies have proven the presence of hundreds of bacteria species, or their remains, on paper surfaces [39, 40].

In most water activity-related studies, sodium chloride or glycerol was used to adjust water activity in the culture medium. However, apart from affecting water activity, sodium chloride also changes osmotic pressure, whereas glycerol can be utilized by some microbes as a stress protectant [41]. At the same time, high molecular weight PEG (PEG6000) is used as an arid mimic in research on rhizosphere microbes [42]. We found that PEG200 reduced water activity of the medium effectively, while the PEG2000 and PEG6000 had limited effect on water activity. In addition, PEG200 was not used as carbon source by FDU301, as the concentration of PEG200 in the medium remain unchanged after culturing for 4 h (data not shown). Using PEG200 as the arid simulator, *B. megaterium* FDU301 was isolated from plaque areas on the paper in an old book.

Transcriptional analysis of *B. megaterium* FDU301 revealed that genes in multiple metabolic pathways responded to the arid environment simulated with 15% PEG200. For example, FDU301 utilized compatible solutes to resist the arid condition. Microorganisms often use a variety of compatible solutes to deal with hypertonic conditions, such as proline, ectoine, and glycine-betaine [43]. We found that the transcription of ectoine biosynthesis-related genes, *ectB* and *ectA*, were greatly enhanced under arid condition. Meanwhile, the expression of two sets of *proVWX* operons related to glycine-betaine/proline transport was not significantly upregulated, or even downregulated (Table 2). These were different from many other bacteria [3], indicating that *B.megaterium* FDU301 might use ectoine, rather than glycine-betaine and proline, as the major compatible solute. It is reported that different *Bacillus* spp. used different compatible solute under osmotic stress [44]. The expression and transportation of other potential compatible solutes, such as spermidine and putrescine, were also increased (Additional file 2: Table S1).

Forming spores is a common strategy of bacteria in *Bacillus* to sustain in unfavorable environments. Genes related to sporulation stage II (*spoIIB, spoIIE, spoIIGA*), and SASP-encoding gene *SspD* were upregulated in the simulated arid conditions. The sporulation of *B. subtilis* can be divided into 6 stages, and each stage requires the co-regulation of multiple genes [45]. The sporulation stage II genes (*spoIIB*, *spoIIE*, *spoIIGA*) are controlled by a series of compartment-specific σ factors and responsible for the asymmetric septation [43]. Small, acid-soluble proteins (SASP) is related to the formation and maturation of spores [46]. However, the expression of genes related to further stages in sporulation were not changed significantly of even downregulated (Additional file 2: Table S1). This was consistent with our observation that there was no significant difference in the rate of sporulation of FDU301 in medium with 0, 5, 15% PEG200 after 4 h cultivation (data not show).

On the other side, genes in oxidative phosphorylation pathway (*atpB, atpE, atpF, atpH, atpA, atpG*, *atpD*, *atpC*), as well as genes related to glycolysis (*pgk, tpiA, frmA*) were downregulated significantly in the simulated arid condition (Additional file 5: Table S4). This might reflect the suppression of general metabolism in response to stress, and was consistent with the decrease in the transcription of many ribosome proteins under the arid condition (Additional file 2: Table S1).

Meanwhile, we found that oxidative stress was the major challenge that *B. megaterium* FDU301 faced in PEG200-mediated arid condition. Significant upregulation of *perR*, a regulator of the peroxidative stress response, was seen. In *Bacillus* spp., PerR is a Fe^2+^ containing DNA binding protein that represses the expression of a series of genes [47]. In the presence of H_2_O_2_, *perR* will lose its DNA binding activity, resulting in the expression of many oxidative stress-responsive genes, including itself, another transcription regulator, *fur*, as well as *dps, katE*, and several other related genes [33, 48]. In consistent with that, the transcription of *fur*, *dps*, and *katE* was found to be significantly increased in the simulated arid condition. *Dps* and *katE* encodes DNA protection protein and catalase, respectively. The fact that adding reductive agents (glutathione or ascorbic acid) to the medium, alleviated the effect of arid stress (data not show), further supported that oxidative stress was the key factor in limiting the growth of FDU301 in the simulated arid condition.

In *Bacillus* spp., *fur* encodes a protein that regulated cellular iron uptake and iron carrier biosynthesis [49]. In the oxidative condition, Fe^2+^ is highly deleterious to DNA. Meanwhile, Fe^2+^ is also essential for *perR*-mediated sensing of oxidative stress. *PerR*, *fur* and *dps* are all Fe^2+^-containing protein. Activation of Fur protein usually results in the inhibition of Fe^2+^ uptake. However, we also saw the increased expression of *feoB*, a gene responsible for Fe^2+^ uptake, in the simulated arid condition, suggesting that the concentration of Fe^2+^ was relatively low in cells under such condition. It seems that a delicate balance of Fe^2+^ is very important for the bacteria to survive in such a stressful condition.

Among DEGs, the expression of transcriptional regulatory factor *tipA* increased dramatically (up to 150.12-fold) compared with the control group. In *Streptomyces* spp., *tipA* belongs to the *MerR* transcriptional regulatory family and is induced by thiostrepton to produce two regulatory proteins, TipAL and TipAS [50, 51]. Many other genes in the *MerR* family have been shown to be involved in the regulation of bacteria on a range of stresses, including oxidative stress and metal ions [52]. It is speculated that *tipA* played an important role in the regulation of stress adaptation to arid condition for *B. megaterium* FDU301, though the exact mechanism needs to be further explored.

Interestingly, the transcription of *tipA* was only moderately upregulated, or even downregulated, in the arid stress mediated by sodium chloride or glycerol, respectively (Additional file 7: Figure S3). Apart from *tipA*, it was also noted that in the arid condition mediated by sodium chloride or glycerol, genes related to oxidative phosphorylation and glycolysis were usually upregulated [53–55], unlike the results we observed in *B. megaterium* FDU301 using 15% PEG200. These results suggested that the arid environment simulated by PEG200 was in some way different from that using salt or glycerol, and the bacterial adaptation to them may also be different.

## Conclusions

A strain of *B. megaterium*, FDU301, was isolated from a paper surface and its transcriptional adaptation to the arid condition, simulated with 15% PEG200, was studied. The arid condition caused oxidative stress for FDU301, and the aerobic respiration was inhibited. FDU301 mitigated the oxidative stress by enhancing the expression of genes related to anti-oxidation, iron ion transporters, and transcriptional regulatory factors. The expression of the transcriptional regulator *tipA* increased significantly. Under the simulated arid conditions, FDU301 increased the biosynthesis of compatible solutes, such as ectoine, spermidine and putrescine, and started the process of sporulation. In low concentration of PEG200 (5%), most of these upregulated genes were also upregulated, but to significantly lower level than that in 15% PEG200 medium, whereas the downregulated genes in oxidative phosphorylation and glycolysis pathways were upregulated significantly, suggesting a preparation for the adverse condition. To our knowledge, this is the first study using RNA-seq to analyze the adaption of *B. megaterium* to arid environments.

## Methods

### Isolation and growth of bacteria strain

A sterile polyester fiber swab was wetted with 20 μl wetting solution (0.15 M sodium chloride+ 0.1% Tween 80), and used to wipe gently on colored plaques of a leaflet from an old book. The swab head was then carefully peeled off with sterile tweezers and transferred into 50 ml LB liquid medium. The sample was agitated at 37°C, 200 rpm overnight, before the suspension was spread on LB agar medium with PEG200 (10% w/w). A total of seventeen bacterial colonies were obtained. Then they were transferred to medium with PEG200 (15% w/w), with only one colony survived. To exam its tolerance to PEG200-mediated arid condition, the bacteria was pre-cultured in LB liquid medium at 37 °C, 200 rpm, for about 8 h, before being transferred to LB liquid medium with different concentrations of PEG200 (5–20% w/w), at an inoculation ratio of 5%. The bacteria were further cultured at 37 °C and 200 rpm. *B. megaterium* NBRC15308 was obtained from Forte Cheung Biological Technology (Shanghai, China).

### Extraction of total RNA and RT-qPCR analysis

The total RNA was extracted from bacteria under normal and the simulated arid conditions (15% PEG200 (w/w)) with the improved Trizol (Takara) method [56]. The RNA was reverse-transcribed into cDNA using the PrimeScript^RT^ reagent Kit with gDNA Eraser (Takara). The cDNA was used as template in RT-qPCR with SYBR qPCR Master Mix (Takara), with *gyrB* as the endogenous control. All reactions were carried out in triplicate. The relative quantitative algorithm (2^-△△CT^) was adopted to calculate the transcription levels of DEGs in different treatments [57]. The primers used in the experiment were displayed in Additional file 8 (Table S5).

### Analysis of transcriptomic data

Total RNAs from *B. megaterium* FDU301 of exponential growth phase (4 h) in arid medium (15% PEG200 (w/w)) or normal medium, were used for RNA-Seq library construction and sequencing (Illumina Hiseq2000 platform, Meiji Biotechnology) [58]. To obtain clean reads, the adapter sequences and low quality bases (the sequencing quality value, Q, less than 20) were removed from the raw sequence [58], and the clean reads were aligned with the *B. megaterium* FDU301 genome using Bowtie2 software [59].

The transcriptional profiles of *B. megaterium* FDU301 under normal (L) and arid conditions with 15% PEG200 (w/w) (P) after 4 h of cultivation were compared. Gene expression was quantitatively analyzed via TPM (transcripts per million reads) algorithm [60]. Genes with a fold of change in expression equal or greater than 2 (|log_2_FC| ≥ 1) and adjusted *P*-value (FDR) less than 0.05 (FDR < 0.05) were identified by DESeq2 software [61] and specified as differently expressed. The experiment was carried out in triplicate. Functional annotation was done using the COG [62] and KEGG databases [63]. edgeR software were used to draw the volcano map of gene expression differences [64].

### Statistical analysis

Data from triplicate or more parallel experiments were used to calculate means and standard deviations. Statistical significance between groups was evaluated using student’s t test.

## Supplementary Information

**Additional file 1:** **Figure S1.** RT-qPCR verification. Ten DEGs were randomly selected and their transcriptional level were determined with RT-qPCR. FC: fold of change in the transcriptional level in simulated arid condition (LB medium with 15% PEG200, w/w) comparing to normal LB medium.

**Additional file 2: Table S1.** Significant DEGs.

**Additional file 3: Table S2.** COG annotation.

**Additional file 4: Table S3.** ABC transporters-related genes.

**Additional file 5: Table S4.** Respiration-related genes.

**Additional file 6:** **Figure S2.** Effect of different arid conditions on the level of ROS in the cell of *B. megaterium* FDU301. The Bacterial ROS Hi-Fluo Assay Kit (Chundubio, China) was used to determine the oxidative stress of FDU301 under different concentrations of PEG200 (0–15% (w/w)).

**Additional file 7:** **Figure S3.** Effect of different water activity regulators on the expression of *tipA* gene. The expression of *tipA* gene in *B. megaterium* FDU301 grown in LB medium of aw 0.985, using PEG200, sodium chloride and glycerol to adjust the water activity, respectively, were determined by real-time RT-qPCR. FC: fold of change in the transcriptional level in arid condition medium comparing to normal LB medium.

**Additional file 8:** **Table S5.** Primers used in this study.

## Data Availability

The datasets generated during the current study are available in NCBI GenBank (accession numbers CP045267-CP045276) and NCBI SRA (accession numbers PRJNA649685).

## Abbreviations

PEG200: polyethylene glycol 200
DEGs: differentially expressed genes
TPM: transcripts per million reads
FDR: false discovery rate
ROS: reactive oxygen species
SASP: small acid-soluble spore proteins
