# Supplementary material for: Transcriptional response of Bacillus megaterium FDU301 to PEG200-mediated arid stress
Source: BMC Microbiol. 2020 Nov 16;20:351. doi: 10.1186/s12866-020-02039-4 (PMC7670681; doi:10.1186/s12866-020-02039-4)
Supplement: Supplementary file 1 — Additional file 1: Figure S1. RT-qPCR verification. Ten DEGs were randomly selected and their transcriptional level were determined with RT-qPCR. FC: fold of change in the transcriptional level in simulated arid condition (LB medium with 15% PEG200, w/w) comparing to normal LB medium. [file 12866_2020_2039_MOESM1_ESM.pdf]

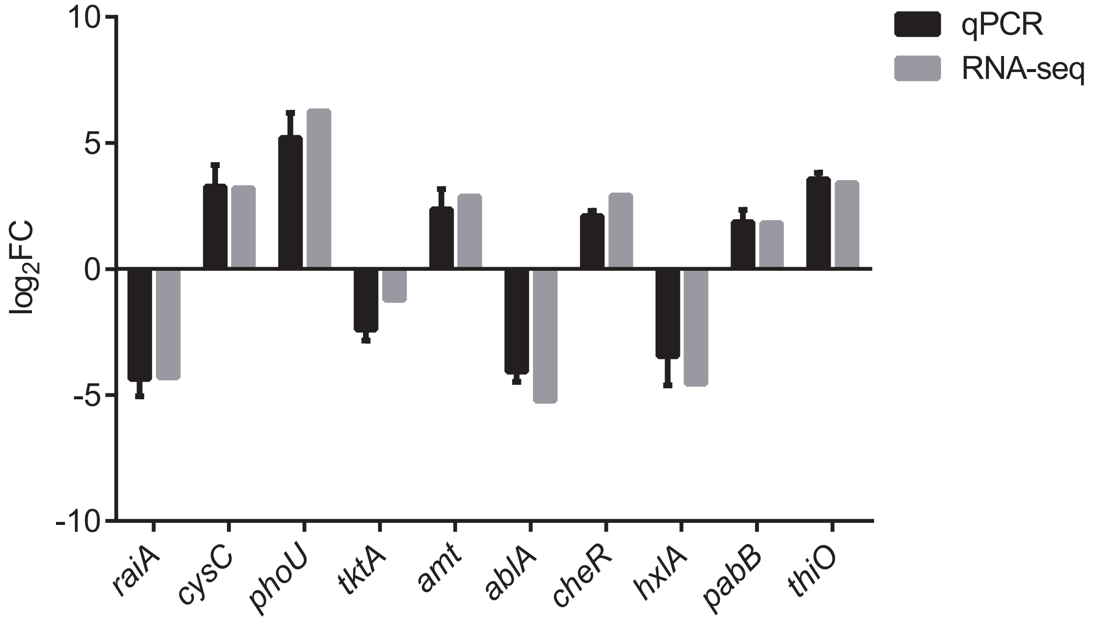

Fig. S1 RT-qPCR verification. Ten DEGs were randomly selected and their transcriptional level were determined with RT-qPCR. FC: fold of change in the transcriptional level in simulated arid condition (LB medium with 15% PEG200, w/w) comparing to normal LB medium.
