# Supplementary material for: Transcriptional response of Bacillus megaterium FDU301 to PEG200-mediated arid stress
Source: BMC Microbiol. 2020 Nov 16;20:351. doi: 10.1186/s12866-020-02039-4 (PMC7670681; doi:10.1186/s12866-020-02039-4)
Supplement: Supplementary file 6 — Additional file 6: Figure S2. Effect of different arid conditions on the level of ROS in the cell of B. megaterium FDU301. The Bacterial ROS Hi-Fluo Assay Kit (Chundubio, China) was used to determine the oxidative stress of FDU301 under different concentrations of PEG200 (0–15% (w/w)). [file 12866_2020_2039_MOESM6_ESM.pdf]

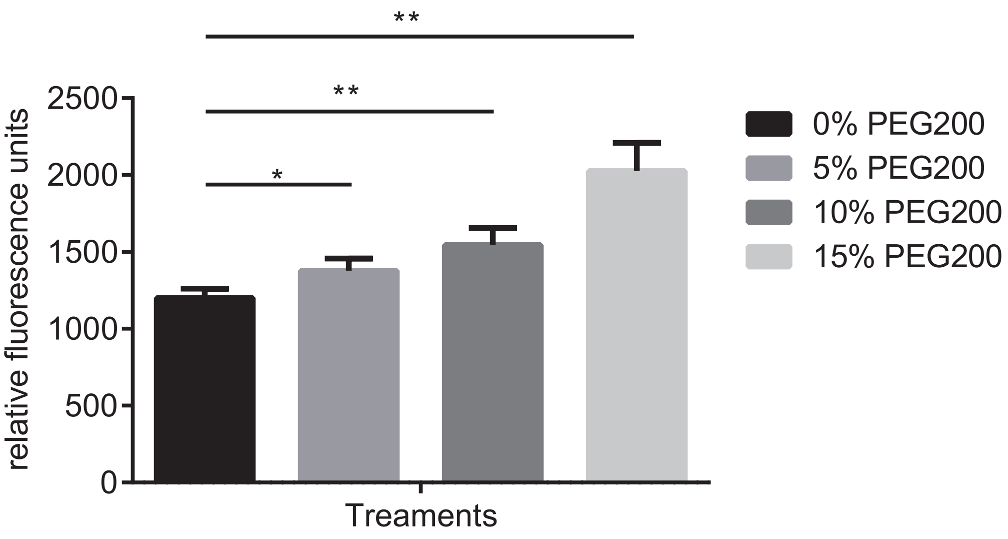


**Fig. S2 Effect of different arid conditions on the level of ROS in the cell of *B. megaterium* FDU301.** The Bacterial ROS Hi-Fluo Assay Kit (Chundubio, China) was used to determine the oxidative stress of FDU301 under different concentrations of PEG200 (0%-15% (w/w)).
