# Supplementary material for: Transcriptional response of Bacillus megaterium FDU301 to PEG200-mediated arid stress
Source: BMC Microbiol. 2020 Nov 16;20:351. doi: 10.1186/s12866-020-02039-4 (PMC7670681; doi:10.1186/s12866-020-02039-4)
Supplement: Supplementary file 7 — Additional file 7: Figure S3. Effect of different water activity regulators on the expression of tipA gene. The expression of tipA gene in B. megaterium FDU301 grown in LB medium of aw 0.985, using PEG200, sodium chloride and glycerol to adjust the water activity, respectively, were determined by real-time RT-qPCR. FC: fold of change in the transcriptional level in arid condition medium comparing to normal LB medium. [file 12866_2020_2039_MOESM7_ESM.pdf]

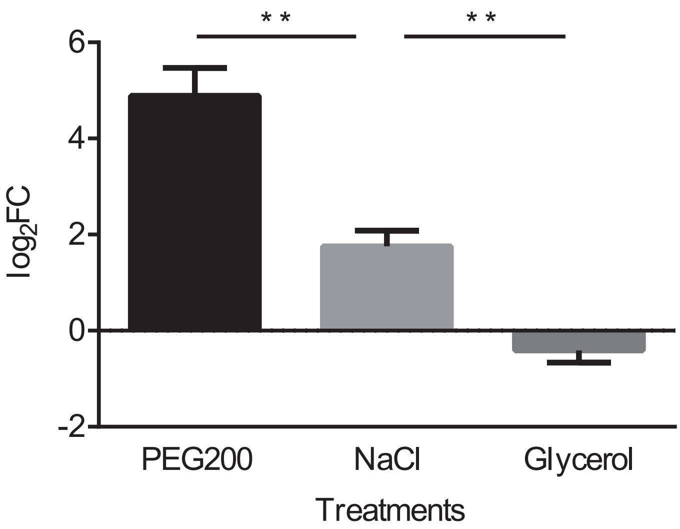

Fig. S3 Effect of different water activity regulators on the expression of *tipA* gene. The expression of *tipA* gene in *B. megaterium* FDU301 grown in LB medium of aw 0.985, using PEG200, sodium chloride and glycerol to adjust the water activity, respectively, were determined by real-time RT-qPCR. FC: fold of change in the transcriptional level in arid condition medium comparing to normal LB medium.
