# Supplementary material for: Transcriptional response of Bacillus megaterium FDU301 to PEG200-mediated arid stress
Source: BMC Microbiol. 2020 Nov 16;20:351. doi: 10.1186/s12866-020-02039-4 (PMC7670681; doi:10.1186/s12866-020-02039-4)
Supplement: Supplementary file 8 — Additional file 8: Table S5. Primers used in this study. [file 12866_2020_2039_MOESM8_ESM.docx]

Table S5 Primers used in this study

| **Gene ID** | **Gene Name** | **Sequences of Forward and Backward Primers (5’🡪3’)** |
| --- | --- | --- |
| Reference gene | *gyrB* | AAGATCCTTCTATTAGCGAAC  AAATTTTATCTAAACGGGCTT |
| FDZ14_RS01625 | *raiA* | GTATCTGTTCGCAGCATTAG  AGCCTCTTCACTATCCATTG |
| FDZ14_RS02510 | *cysC* | TGTAGACAGCGGTCAAGT  ACATTCTTCTAACGGACACT |
| FDZ14_RS04590 | *phoU* | CGTGGATGACTTAGAAGAAGA  CTGCCATACGCTCAACAT |
| FDZ14_RS10735 | *tktA* | ACGGCAATGACTTGACAG  ATCGGAACCTAATGGAGAAC |
| FDZ14_RS14160 | *amt* | GGTTCGGCAGTTATCCAT  GCACTTAGCGTACTTCCT |
| FDZ14_RS15690 | *ablA* | TGGTTAATGGATGAGAACGA  CGGCAGTACATAGAGCAT |
| FDZ14_RS20245 | *cheR* | ACCGATATGAACGAAGATGT  AGTGATGGTCCGTCTGATA |
| FDZ14_RS23355 | *hxlA* | CACAGGATACGACCTTCAA  CCGCCAACGATAACAAGA |
| FDZ14_RS27425 | *pabB* | ATGTGATGCTTGTTGACTTG  CCAGTAATCGTTCCTCCAG |
| FDZ14_RS25100 | *thiO* | GCTCTTCTATCGCTTATCAT  TTAGTTCCTTCACCAGTTCA |
| FDZ14_RS25760 | *perR* | TACTGGCGTTCGTATCAC  TCACCGTATGTTAGTTCCTT |
| FDZ14_RS05160 | *fur* | GCGAATTAAGCAGCAGTT  CTTGACGAGGAGATATACATC |
| FDZ14_RS11995 | *dps* | CAAACGAGTGAAGAAATGGT  ATGTTGCCGATTGTTCCA |
| FDZ14_RS00915 | *katE* | GCTACATTCTCACCTGGAA  CCGTCACGCTGATAGTTAT |
| FDZ14_RS23330 | *feoB* | GGCGACACAATGAAGACA  CGAGATAAGCGACAACCAA |
| FDZ14_RS06830 | *ectB* | AACTGGAGGAGACGCAAT  TACCGCTGATGCTTAACG |
| FDZ14_RS06845 | *ectA* | ATTGACGGTGTGCCTATG  AACTAAGAAGGAGCGGATAA |
| FDZ14_RS03790 | *spoIIB* | TTACATTGTTCAAGCAGGTG  CCAACGACAAGAGAATAGGT |
| FDZ14_RS27465 | *spoIIE* | AATCGGTTCTACGCCAAG  AATACGCCATCACTCATCAT |
| FDZ14_RS05795 | *spoIIGA* | CAGTTGCTGCTTGCTCTA  GCTTCGGATGAACAATACAC |
| FDZ14_RS21810 | *sspD* | CTATTAACACCAGGTGTAGAAC  TTCTCCGCCTACTGAACC |
| FDZ14_RS11440 | *tipA* | GTCACCATCCTCTACAGAAG  CGTAGCGATTGATGTTAGTT |
| FDZ14_RS01350 | *atpB* | TTGACGCTAGGTGTAACTT  CAACTACCATAACGGCTAAC |
| FDZ14_RS01355 | *atpE* | ATCGTGGTTGTTGTCGTAT  AGTTGGCACTGCTAATAGAA |
| FDZ14_RS01360 | *atpF* | GTGATGTTCCTTATCCTACTTG  GCTTCTTCGCTTCCTCAT |
| FDZ14_RS01365 | *atpH* | TTCCTAGCGAACGAAGTAC  GCCATCATAGATACGATTACC |
| FDZ14_RS01370 | *atpA* | GCTGGAGAACTGGTTGAAT  CTACTGGCTGACCTAATGAG |
| FDZ14_RS01375 | *atpG* | AAGTAGTCTCAAGCGTAGC  GATGGCGTTCTTCAATAGC |
| FDZ14_RS01380 | *atpD* | CGTAGCATATCCGTTCCTAA  TACCAGCACCAGCAATCT |
| FDZ14_RS01385 | *atpC* | GTGTAGTGACTCCTGATGG  AGTTCTGTACTGCTTGCTT |
| FDZ14_RS01875 | *pgk* | AACATATTCCAGCAGTAGCA  AGTCCTCCACCGATGATTA |
| FDZ14_RS01880 | *tpiA* | ACGAAGAGTACGAACAAGAT  AACGCTACGGATGTATGC |
| FDZ14_RS16380 | *frmA* | AGGACAGGCTGAACTATTAC  AATAAGCGGTTGGCAGAA |
